# Venous thromboembolism treatment failure during use of factor Xa inhibitors—association with thoracic outlet syndrome and development of chronic thromboembolic pulmonary hypertension

**DOI:** 10.1080/07853890.2024.2404549

**Published:** 2024-12-03

**Authors:** Matti Kaksonen, Piia Simonen, Riitta Lassila, Markku Pentikäinen

**Affiliations:** a Heart and Lung Center, Cardiology, Helsinki University Hospital and University of Helsinki, Helsinki, Finland; b Department of Hematology, Coagulation Disorders Unit, Comprehensive Cancer Center, Helsinki University Hospital, Helsinki, Finland; c Research Program in Systems Oncology, Faculty of Medicine, Helsinki University, Helsinki, Finland

**Keywords:** Venous thromboembolism, chronic thromboembolic pulmonary hypertension, thoracic outlet syndrome, factor Xa inhibitor

## Abstract

**Background:**

Factor Xa inhibitors (FXaI) are recommended for treatment of venous thromboembolism (VTE). However, in FXaI trials there is a 2–3% treatment failure rate. This observational study aimed to elucidate factors associated with recurrent VTE during coagulation FXaI treatment.

**Methods:**

Ten consecutive FXaI failure cases were included. Various thrombosis risk scores were assessed, thrombophilia was screened, and coagulation activity was followed-up, to tailor individual anticoagulation strategies.

**Results:**

Our patients were young (mean age 37.5 years, range 22–55), six being women. Index VTE was pulmonary embolism (PE) in eight patients, and upon recurrent PE, six of them developed chronic thromboembolic pulmonary hypertension (CTEPH). Although initially many patients appeared to have unprovoked VTE, all had major VTE risk factors. Seven patients had chronic venous obstruction: five subclavian (thoracic outlet syndrome, TOS) even though only two had upper extremity deep vein thrombosis at index thrombosis, plus one common iliac, and one with chronic paraplegia. Five patients had multiple VTE risk factors and four had thrombophilia. VTE risk scores varied from the lowest (TOS patients) to the highest risk (multiple risk factors/thrombophilia). FXaI failure occurred on average at 97 days of therapy (range 15–279) without evident noncompliance. D-dimer levels declined from the index thrombosis to FXaI failure, and re-thrombosis resisted further anticoagulation, low D-dimer referring to impaired fibrinolysis. The majority (8/10) of patients required mechanical/surgical interventions.

**Conclusions:**

Our results underline careful risk assessment upon PE and reoccurrence, with inclusion of TOS as a risk factor of VTE and CTEPH.

## KEY MESSAGES

Failure of factor Xa inhibitors (FXaI) to treat venous thromboembolism in our young patient cohort led to chronic thrombosis requiring mechanical intervention

Thoracic outlet syndrome (TOS) was the most common cause of venous obstruction

The development of chronic thrombosis in young patients calls upon careful assessment of risk factors, suitability of FXaI, and recognizing venous obstruction, notably TOS, as a possible etiologic factor

## Introduction

Venous thromboembolism (VTE), clinically presenting as pulmonary embolism (PE) or deep vein thrombosis (DVT), is a major burden to patients and healthcare systems [1]. VTE is estimated to be the third most common vascular disorder after acute coronary syndromes and stroke [2]. VTE requires anticoagulant treatment to support the endogenous fibrinolytic system to dissolve the index thrombosis and to prevent future recurrence and complications. A rare complication of acute PE, but the most severe, is chronic thromboembolic pulmonary hypertension (CTEPH), which occurs in acute symptomatic PE in about 2–4% of cases [3].

Traditionally, warfarin, a vitamin K antagonist (VKA), has been the drug of choice in VTE until the approval of direct thrombin inhibitor, dabigatran, and the coagulation Factor (F) Xa inhibitors (FXaI) apixaban, edoxaban and rivaroxaban. Compared with warfarin, thrombin and FXa inhibitors have initial drug-specific dosing schemes with or without low-molecular-weight heparin (LMWH). Moreover, they share comparable efficacy, have more predictable pharmacokinetics and some, but notable, interactions, and lower bleeding risks than VKAs [4, 5].

European Society of Cardiology guidelines recommend FXa or thrombin inhibitors as the first line of oral antithrombotic therapy for treatment of acute PE and DVT [6, 7]. Intermediate- to high-risk PE patients are advised to be treated with LMWH for the first 2–3 days to ensure that they reach clinical stability before changing to an oral anticoagulant. The use of unfractionated heparin (UFH) in acute PE patients is recommended in more complicated situations such as overt hemodynamic instability, serious renal impairment (creatinine clearance ≤30 mL/min) or severe obesity [6]. Accordingly in the outpatient setting, overall prescriptions of VKAs have significantly decreased [4]. Recurrent VTEs despite FXa or thrombin inhibitor treatment have occurred in 2–3% of cases in large clinical trials [8–11]. These recurrent VTEs, despite adequate FXaI treatment, are called FXaI failures. Cases of FXaI failure represent a clinically challenging patient group [12].

## Methods

Young VTE patients, with unsatisfactory FXaI treatment results, have been encountered at Helsinki University Hospital (HUH). FXaI treatment had not dissolved the index thrombosis, which progressed, or a new thrombosis was diagnosed. We retrospectively studied 10 of these consecutive FXaI failure cases during a 6-year period. The patients were admitted to HUH for treatment of VTE through consultations with cardiology and coagulation disorder units in 2015–2020. Some of the cases had been initially managed in other healthcare facilities in Finland and the patients were referred to the tertiary center of HUH due to lack of efficacy of anticoagulation therapy with FXaIs. Observational data on these patients were retrospectively collected from electronic medical records covering a total cohort of 4050 PE patients who were treated in HUH during those years. The aim of this study was to elucidate factors which predispose patients to FXaI failure, and its clinical consequences.

Recurrent VTE was defined as a new or progressing PE or DVT detected after initial thrombosis. FXaI failure was here defined as diagnosis of a recurrent VTE during FXaI treatment and/or lack of efficacy to dissolve the index thrombosis in patients compliant with the treatment. Index and failure cases of VTE were analyzed for their type, provoked and unprovoked thrombotic risk factors, presence of chronic venous obstruction, antithrombotic medication prior to FXaI treatment, timing of FXaI failure, the type and dose of FXaI used, possible right ventricle strain, and other diagnoses and outcomes after FXaI failure. The patients were assessed by using three VTE risk scores: Padua (≥4 high risk of VTE) [13], Caprini (0 lowest, 1–2 low, 3–4 moderate, 5–8 high, ≥9 highest risk of VTE) [14, 15], and a local HUH guideline for thromboprophylaxis (<3 low, 3 moderate, >3 high thrombosis risk) [16]. In addition, Pulmonary Embolism Severity Index (PESI) scores of risk classes (I, very low: ≤65 points; II, low: 66–85 points; III, intermediate: 86–105 points; IV, high: 106–125 points; V, very high: ≥126 points) [17] at the time of FXaI failure, and the clinical CTEPH risk score (> 6 high risk) [18] were assessed during index thrombosis. Patient compliance, as assessed by purchases of FXaIs from pharmacies was verified from the National Prescription Centre of Finland (Kela).

Laboratory results from blood samples were collected to cover three timepoints: the index thrombosis, the start of FXaI treatment, and at the detection of FXaI failure. Laboratory assessments covered N-terminal B-type natriuretic peptide (proBNP), lipids, routine clinical chemistry, complete blood cell count, and coagulation screening for prothrombin time (PT), international normalized ratio (INR), activated partial thromboplastin time (aPTT), thrombin time (TT), fibrinogen, antithrombin, factor VIII coagulation activity (FVIII) and D-dimer. All patients were screened for thrombophilia by analyzing mutations of factor V (FV) Leiden and prothrombin genes, levels of natural anticoagulants antithrombin, protein C activity and protein S free antigen, and phospholipid antibodies against beta-2-glycoprotein I, cardiolipin as well as lupus anticoagulant.

Laboratory methods for analyzing the blood samples were the following: A Sysmex® XN-9000 haematology analyzer (Kobe, Japan) for K2-EDTA tubes was used for complete blood counts. C-reactive protein (CRP) and creatinine (plus the estimated glomerular filtration rate (eGFR) in the EPI formula), were analyzed with a Siemens Atellica® Solution chemistry analyzer (Siemens Healthineers). Citrated plasma (3.2% Na-citrate, Becton Dickinson, New Jersey, USA) was collected for assessment of coagulation times and factors. Coagulation tests were performed using ACL TOP® 500 and 750 analyzers (Instrumentation Laboratory, Naples, Italy) with the following reagents: PT with Owren’s PT (Medirox®, Nyköping, Sweden), aPTT, FVIII activity, fibrinogen with the Clauss method, antithrombin activity, and D-dimer concentrations (D dimer HS 500, FEU units) all with HaemosIL® reagents (Instrumentation Laboratory, Naples Italy). Factor V and prothrombin gene mutations were detected by cyclic minisequencing. Protein C activity was measured using photometry testing, whereas protein S free antigen was analyzed with photometric and immunochemical assays. Lupus anticoagulant was assessed by means of phospholipid-sensitive aPTT as well as the Russel viper venom test. Immunoglobulin G antibodies against cardiolipin and beta-2-glycoprotein I were detected by enzyme immunoassays.

### Statistical analysis

The laboratory data were tested for normal distribution by using the Shapiro–Wilk test. Accordingly, differences between groups were studied by using One-way ANOVA and Kruskal–Wallis tests with Mann–Whitney U post-hoc tests. A p-value of <0.05 was considered statistically significant.

### Ethics approval

The study protocol was approved by the Institutional Review Board of Heart and Lung Center, Helsinki University Hospital, Helsinki, Finland (3/2018). As this was an observational retrospective study, ethical board approval and informed consent were waived by the Institutional Review Board of Heart and Lung Center, Helsinki University Hospital, Helsinki, Finland (3/2018) based on Guidelines for ethical review in human sciences by Finnish National Board on Research Integrity (TENK). The individual patient data were anonymized at the time of data collection.

## Results

### Demographics and index thrombosis

Our patients were young, their average age being 37.5 years (Table 1). Six were women, of two being on oral contraceptives. Only one patient was obese (body mass index (BMI) over 30 kg/m^2^), while their average BMI was 25 kg/m^2^. Seven of the patients had obvious VTE-predisposing comorbidities. Two patients had a history of VTE, and five had a family history of thrombosis. Four patients had thrombophilia, of whom one had combined thrombophilic traits; one prothrombin mutation G20210A and two cases of antiphospholipid antibody syndromes were diagnosed. Two patients were current smokers. None of the patients was diagnosed with cancer. Mild hyperlipidemia was noted in two patients. In seven patients chronic vein obstruction was observed, often late.

**Table 1.** Demographics of the patients.

Age, years	
Mean (SD)	37.5 (11.4)
Median (IQR)	35 (15.5)
Sex female	
n (%)	6 (60%)
BMI (kg/m2)	
Mean (SD)	25.1 (6.8)
Median (IQR)	24 (3.1)
Comorbidities*	
None, n (%)	3 (30%)
Thrombophilia#	
n (%)	4 (40%)
Family history of thrombosis	
n (%)	5 (50%)
Oral contraception	
n (%)	2 (20%)
Smoking	
n (%)	2 (20%)
Chronic vein obstruction°	
n (%)	7 (70%)

*Comorbidities included hypertrophic cardiomyopathy with implantable cardioverter defibrillator pacemaker, migraine, dyslipidemia, splenectomy, asthma, chronic obstructive pulmonary disease, bipolar disorder, previous venous thromboembolism, sleep apnea, and ankylosing spondylitis.

^#^Thrombophilia included high coagulation factor VIII, lupus anticoagulant and heterozygotic prothrombin gene mutation, type 1 protein C deficiency, and beta-2-glycoprotein I and cardiolipin antibodies.

°Chronic vein obstruction included 5 thoracic outlet syndrome (50%), paraplegia, and May-Thurner syndrome.

BMI, body mass index; SD, standard deviation; IQR, interquartile range.

As for their index thromboses, seven patients had PE without DVT, one had DVT, one upper extremity DVT (UEDVT) and one patient had both PE and UEDVT (Table 2). In our patient series of FXaI failures, eight patients had been treated with rivaroxaban and two patients with apixaban. In five patients LMWH had been commenced for a couple of days before FXaI initiation, but in two cases warfarin had been used with an unstable INR for about 30 days before the start of FXaI treatment. Two patients had received local catheter-based UEDVT thrombolysis with alteplase before FXaI treatment. Overall, although being young, eventually all patients had major risk factors of VTE.

**Table 2.** Index thrombosis.

Patient	Thrombosis type	Anticoagulation prior to FXaI	Dose of FXaI at the time of failure
1	PE and UEDVT	LMWH	Rivaroxaban 20 mg x1
2	PE	LMWH, warfarin	Rivaroxaban 20 mg x1
3	PE	No	Rivaroxaban 20 mg x1
4	PE	No	Rivaroxaban 20 mg x1
5	PE	No	Rivaroxaban 20 mg x1
6	DVT	LMWH	Rivaroxaban 15 mg x2
7	PE	LMWH, warfarin	Rivaroxaban 20 mg x1
8	UEDVT	LMWH	Rivaroxaban 20 mg x1
9	PE	LMWH	Apixaban 5 mg x2
10	PE	LMWH	Apixaban 5 mg x2

FXaI coagulation, Factor Xa inhibitor; PE, pulmonary embolism; UEDVT, upper extremity deep vein thrombosis; LMWH, low-molecular-weight heparin; DVT, deep vein thrombosis.

### FXaI treatment failure leading to recurrent thrombosis

The median time to diagnosis of FXaI treatment failure was 74 days (mean 97, range 15–279) (Table 3). We were able to verify the FXaI purchases, and there was no evidence of efficacy-impairing drug-to-drug interactions or noncompliance. In seven patients, treatment failure was diagnosed during once-daily administered rivaroxaban, and in one earlier, at the twice-daily dosing period (Table 2). Six patients were found to have acute thrombosis risk factors before or during FXaI treatment (Table 3). The thrombosis coinciding with the FXaI failure was of the same type as the index thrombosis in all except one patient, whose index VTE was PE, and FXaI failure was associated with DVT. Upon the FXaI failure, seven patients were diagnosed with a new PE, two patients with DVT, and one with a new UEDVT (Table 3). At presentation, all seven cases of PE showed PESI scores in the lowest risk category (class 1). However, six patients suffered from right-sided heart strain revealed by echocardiography, and six patients had elevated levels of proBNP or BNP at the time of treatment failure.

**Table 3.** Thrombosis at FXaI failure.

Patient	Time to failure (days)	Acute risk factors	Site of thrombosis	PESI score	Right heart strain	proBNP (ng/L)
1	64	Atraumatic rib fractures due to severe cough	PE	36	Yes	2500
2	28	Sepsis, endocarditis, surgical change of ICD, immobilization, oral contraception	PE	46	No	429
3	170	No	PE	39	Yes	12
4	79	Pneumonia	PE	42	Yes	2310
5	122	2 days intermission of FXaI*, oral contraception, long flight	PE	41	Yes	54
6	15	Pilonidal cyst and ankle surgery, immobilization	DVT	NA	No	33
7	16	No	DVT	NA	No	200
8	131	No	UEDVT	NA	No	no
9	69	No	PE	56	Yes	BNP 239
10	279	Viral pneumonia	PE	26	Yes	702

FXaI coagulation Factor Xa inhibitor, PESI pulmonary embolism severity index and risk classes I (Very low): Points ≤65, II (Low): Points 66–85, III (Intermediate): Points 86–105, IV (High): Points 106–125, V (Very high): Points ≥126), NA not applicable), BNP B-type natriuretic peptide (proBNP normal reference values: men, age under 50 years <84 ng/L, women under 50 years <155 ng/L, men aged 50–65 < 194 ng/L, women aged 50–65 < 222 ng/L; BNP normal reference level <100 ng/L), PE pulmonary embolism, ICD implantable cardioverter defibrillator, *due to womb thermo-ablation, DVT deep vein thrombosis, UEDVT upper extremity deep vein thrombosis.

### Management of FXaI failure

**Figure 1.**
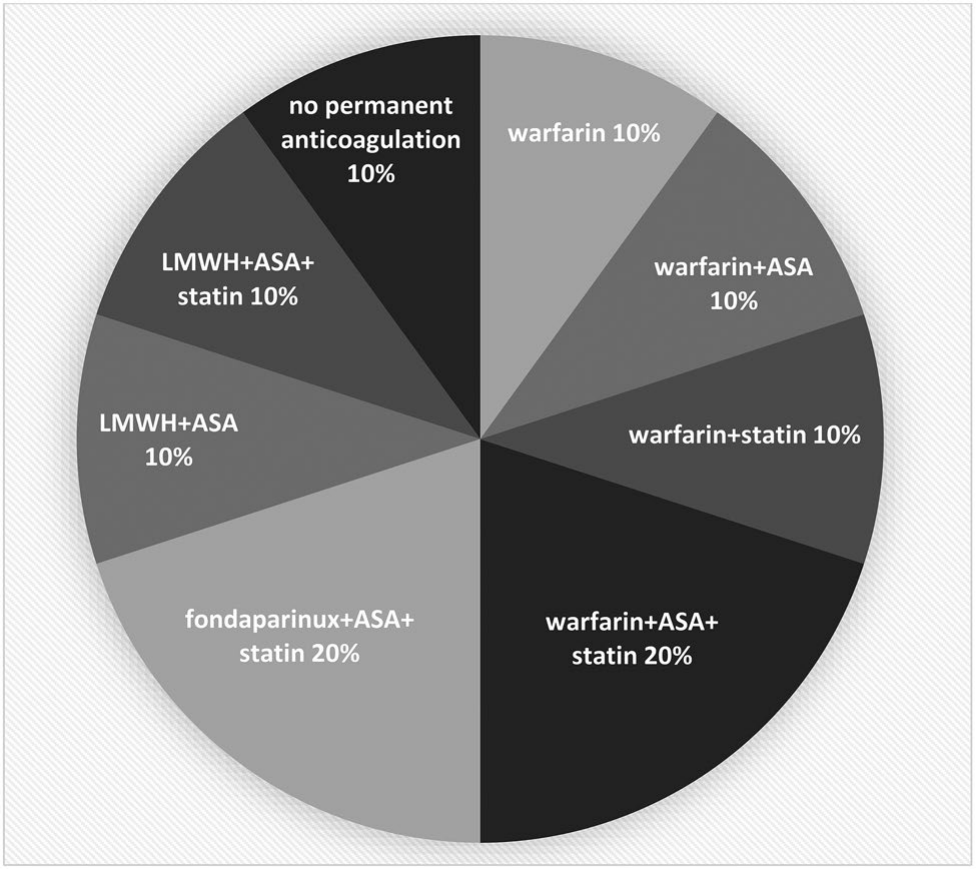
Long-term antithrombotic treatment. Long-term antithrombotic treatment was individually tailored to each of the 10 patients. The use of different treatment combinations is shown. In one patient without CTEPH antithrombotic treatment was discontinued after operation for TOS. LMWH; low-molecular-weight heparin, ASA; acetylsalicylic acid, CTEPH; chronic thromboembolic pulmonary hypertension, TOS; thoracic outlet syndrome.

The antithrombotic combination therapies were individually tailored to prevent further thrombosis in both the acute and permanent treatment phases (Table 4, Figure 1). In the acute phase, recurrent thrombosis could be prevented by switching to tailored antithrombotics in nine patients, and the anticoagulant was in many cases supplemented with acetylsalicylic acid (ASA) (Table 4). In majority of the patients, parenteral antithrombotic treatment was commenced to control the progressing thrombosis. As for continued and seemingly permanent antithrombotic treatment, five patients were switched to warfarin, two continued with LMWH sc, and two with fondaparinux sc (Figure 1). In addition to their anticoagulant treatment, due to the exceptional thrombotic burden, seven patients were also co-administered ASA, and statin was administered to six patients [19]. In one patient without CTEPH, antithrombotic treatment was discontinued after thoracic outlet syndrome (TOS) had been successfully treated by means of surgery and percutaneous transluminal angioplasty.

**Table 4.** Antithrombotic treatment upon FXaI failure.

Patient	Antithrombotics to prevent further thrombosis
1	Fondaparinux + ASA + statin, LMWH + ASA + statin
2	LMWH + ASA
3	LMWH + ASA, argatroban + ASA, fondaparinux + ASA + statin, LMWH + ASA + statin
4	Fondaparinux + ASA + statin, argatroban + statin
5	LMWH, warfarin
6	LMWH + clopidogrel
7	Warfarin, warfarin + ASA, LMWH + ASA + statin
8	Antithrombotic treatment was unable to prevent further thrombosis
9	Fondaparinux
10	Warfarin

These medications prevented further thrombosis until possible mechanical procedures. FXaI coagulation, Factor Xa inhibitor; ASA, acetylsalicylic acid; LMWH, low-molecular-weight heparin.

Eight patients received further vascular diagnoses after the failed treatment with FXaI, such as TOS, May–Thurner syndrome, or cerebral infarction, and six of them were diagnosed with CTEPH. Strikingly, 4/6 CTEPH patients were also diagnosed with TOS. Eight patients required mechanical and/or angiological treatment to manage their thrombosis. Altogether, three patients were treated by means of thrombectomy, six patients by means of pulmonary endarterectomy surgery, and three by means of balloon pulmonary angioplasty. After these management efforts all patients remain alive and clinically stable.

### Clinical risk scores

According to clinical risk factor scoring, high VTE risk scores were observed in four patients when using both Padua and Caprini scores, and five patients showed high HUH scores during FXaI treatment (Table 5). High mean risks were observed when using Caprini and HUH scores. The CTEPH risk score was high in two patients, but the mean CTEPH risk score was low despite the frequent need for invasive procedures in our series. Thus, all our patients carried multiple risk factors of thrombosis, but in only half of them VTE risk scores were clearly elevated.

**Table 5.** Padua, Caprini, and HUH VTE risk scores during FXaI treatment and CTEPH risk score during the index thrombosis.

Patient	Padua	Caprini	HUH	CTEPH
1	2	0	0	6
2	7	5	8	3
3	0	0	0	9
4	1	0	6	6
5	1	2	1	5
6	6	12	13	0
7	6	12	11	2
8	0	0	0	0
9	1	4	3	11
10	8	15	13	2
Mean	3.2	5	5.5	4.4

Padua (≥4 high risk of VTE), Caprini (0 lowest, 1–2 low, 3–4 moderate, 5–8 high, ≥9 highest risk of VTE), and HUH (Helsinki University Hospital guideline for thromboprophylaxis, <3 low, 3 moderate, >3 high thrombosis risk) VTE risk scores. CTEPH risk score (chronic thromboembolic pulmonary hypertension, >6 is a high-risk score). FXaI coagulation, Factor Xa inhibitor.

## Laboratory assessment

### Index thrombosis

**Figure 2.**
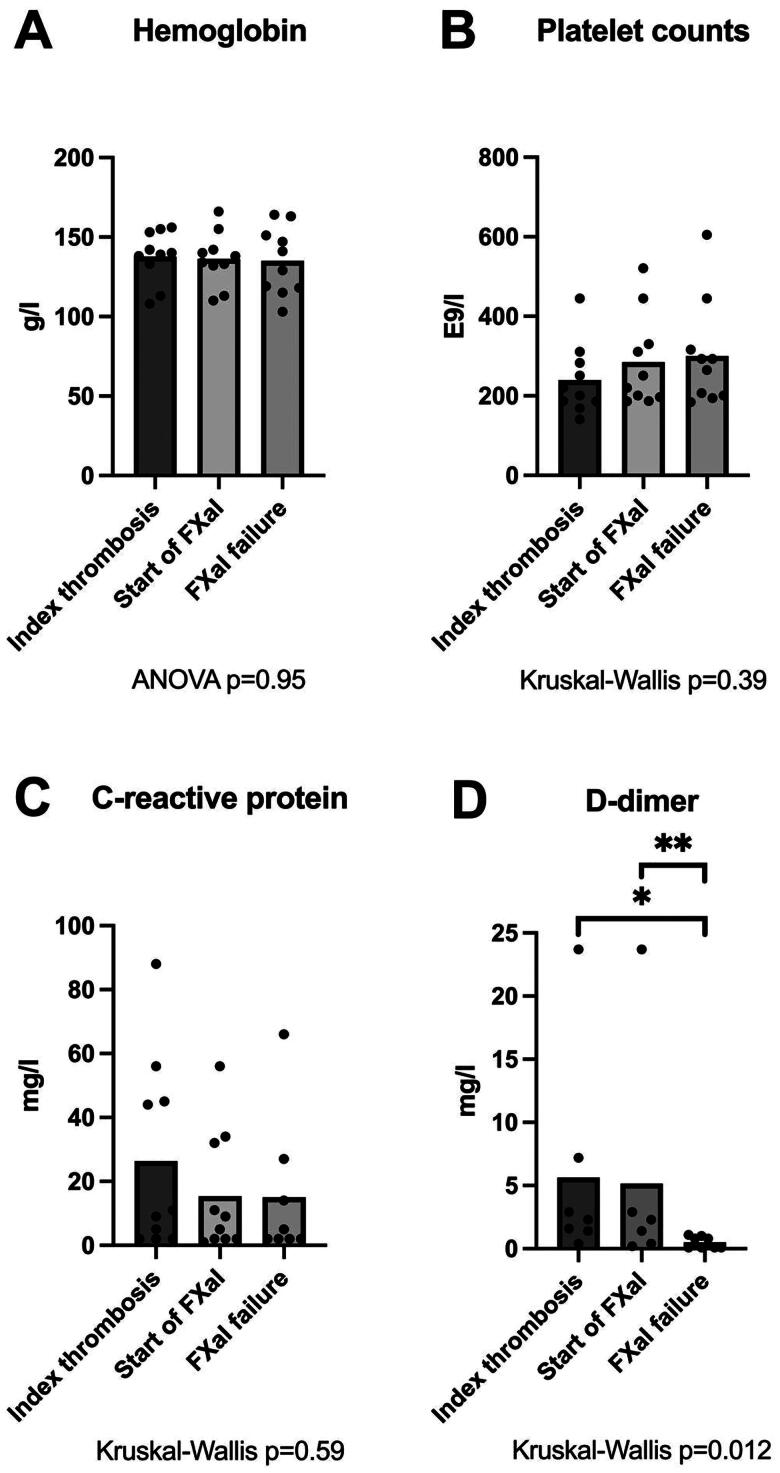
Evolution of hemoglobin (a), platelet count (b), C-reactive protein (CRP) (c), and D-dimer (d) from index thrombosis to FxaI failure. *p < 0.01, **p < 0.05, FxaI;coagulation factor Xa inhibitor.

At the index thrombosis, five patients had no anticoagulation, three patients were on LMWH, and two patients were on FXaI treatment. According to eGFR and alanine transaminase (ALT) results, renal and liver function were normal (data not shown). Two patients had persisting mild anemia of unknown origin (Figure 2(a)). Levels of CRP were clearly elevated in four patients (Figure 2(c)). One patient had a spontaneously prolonged PT value (PT 50%), and another had a markedly elevated FVIII level (456 IU/dL). In only three patients was aPTT measured, and the values were within the reference range. TT was measured in six patients, and all values were within the normal range, indicating normal fibrinogen function. Of the D-dimer values (available in 7/10 patients) one was within the normal range (at 0.4 mg/L), while the others varied (Figure 2(d)).

### FXaI initiation

The mean time between the index thrombosis and FXaI initiation was eight days (median three, range 0–35). At the initiation of FXaI treatment, six patients were on LMWH treatment, three were without anticoagulation treatment, and one was on warfarin. Two patients continued to have mild anemia (Figure 2(a)). In addition, two patients had thrombocytosis and three had clearly elevated CRP values (Figure 2(b,c)). Prolonged PT/high INRs (PT 57 and 63%, and INR 7.3 with warfarin) were observed in three patients before FXaI treatment and two of them had coinciding anemia (Figure 2(a)). Levels of D-dimer were available in six cases, being high in only one patient (23.7 mg/L), but only moderately elevated in three.

### FXaI treatment failure

Eight of the patients were on FXaI treatment at the time of blood collection for the coagulation tests, and treatment of two of them had been switched to LMWH. Two patients presented with thrombocytosis, one with former splenectomy due to trauma and the other with reactive thrombocytosis (Figure 2(b)). PT was prolonged in three patients. Three patients had shortened aPTTs (25 or 26s), and two had, also, prolonged PTs. APTT was prolonged in one patient (46s), indicating lupus anticoagulant or the effect of FXaI. Two patients had shortened TTs (both 15s). Two patients had FVIII values exceeding 190 IU/dL (total range 140–297 IU/dL). Fibrinogen levels were elevated in three patients (4.1, 4.4 and 5.9 g/L). Levels of D-dimer were low overall (mean 0.5 mg/L, range 0.5–1.1 mg/L), and in the two patients with slightly elevated D-dimer values (1.0 and 1.1 mg/L), CRP levels were elevated (Figure 2(d)).

### Changes in laboratory values

As compared with the index thrombosis and the start of FXaI treatment, blood coagulation tests were more frequently carried out upon the FXaI failure. Hemoglobin and platelet counts did not differ at the different time points (Figure 2(a,b)). Levels of CRP were modestly lowered (from a mean of 26 to 15 mg/L) (Figure 2(c)). On the other hand, mean D-dimer levels were clearly lowered, to one tenth from the time of the index thrombosis (mean 5.6 mg/L) and the start of FXaI administration (mean 5.2 mg/L), to treatment failure (mean 0.5 mg/L) (Figure 2(d)), compatible with impaired fibrinolytic activity at the time of FXaI failure.

## Discussion

Although FXaIs are successfully used to treat majority of patients with DVT and PE, we found a series of relatively young patients whose VTE thrombosis reoccurred or failed to resolve during the FXaI treatment. Significantly, in most patients of this small case series, this FXaI failure associated with PE and resulted in chronic thromboembolism requiring various invasive interventions. Our patients depicted two risk scenarios: either TOS with some risk factors, or the presence of multiple (≥4) recognizable thrombotic risk factors. The main observation was that in all TOS patients, the venous obstruction was recognized in association with FXaI failure, rather than the index thrombosis, although in two index thrombosis was UEDVT. UEDVT is not included in the indications for FXaIs. However, after market authorization, real-world use widens without always having the inclusion and exclusion criteria of the initial phase-3 studies of the drug in mind.

In our small patient series, the presence of chronic vein obstruction was common (70%). In venous TOS, compression and impingement of the subclavian vein within the costoclavicular space leads to episodic venous obstruction, decreasing blood flow. Repetitive compression injures the subclavian vein, causing progressive scarring, focal stenosis, and eventual thrombosis, referred to as effort thrombosis or Paget–Schroetter syndrome [20, 21]. Three of the cases of TOS were diagnosed in men with a sporting background. Indeed, competitive athletes are known to have a higher predisposition to the development of TOS due to repetitive overhead motion, muscle imbalance and decreased shoulder stability [22]. Also, minor trauma, common in sports, predisposes patients to thrombosis, especially the ones with a genetic predisposition [23]. Other causes of vein obstruction were May–Thurner syndrome and chronic paraplegia. In May–Thurner syndrome, the right common iliac artery compresses the left common iliac vein against the lumbar spine, predisposing the individual to common iliac vein thrombosis [24]. An acute spinal-cord injury is highly associated with a risk of VTE, despite thromboprophylaxis, and acute paraplegia is an independent risk factor of VTE, which alleviates with time [25]. The risk of VTE in cases of paraplegia after one year is 0.2%, and after two years, 0.1%, matching with risk in the general population [26]. Finally, pacemakers induce a hypercoagulable state through endothelial damage, blood flow impairment, and activation of the contact system. The results of one study suggest that clustering of the classical VTE risk factors is associated with thromboembolic pacemaker complications [27]. Treatment with FXaI was initiated to treat PE in one of our patients with a pacemaker. Apparently, due to the high VTE risk-factor burden, including infection, the FXaI failure developed.

Thrombophilia was found in four of our patients. Their thrombophilic traits included a very high FVIII level, type 1 protein C deficiency, positive lupus anticoagulant combined with heterozygotic prothrombin gene mutation, and antiphospholipid syndrome. The protein C deficiency was diagnosed before the index PE, but for unknown reasons former anticoagulation therapy to treat DVT and PE had been stopped. The two antiphospholipid syndrome patients were diagnosed *via* two separate laboratory assays. According to a meta-analysis, thrombin and FXa inhibitor usage is supported in low-risk thrombophilias (i.e. prothrombin gene mutation or high FVIII) without concerns (except for apixaban, due to lack of data) [28]. In contrast, only limited data are available to support the use of thrombin or FXa inhibitors in high-risk thrombophilias (e.g. protein C deficiency) [28]. The European Medicines Agency (EMA) Pharmacovigilance Risk Assessment Committee has stated that: ‘Direct oral anticoagulants are not recommended for patients with a history of thrombosis who are diagnosed with antiphospholipid syndrome’ [29]. According to the current thrombophilia recommendations, only the patient with a high level of FVIII could have been treated with a FXaI at the index thrombosis. However, the majority of thrombophilias in our patients were not diagnosed until FXaI failure occurred. Indeed, diagnosing thrombophilia early reduces the risk of treating the patient with an inappropriate anticoagulant, or discontinuing anticoagulation in cases of combined or severe thrombophilia.

Limited laboratory results were available in connection with the index thrombosis and when starting the FXaI treatment, although the patients were young and initially thrombosis appeared to be idiopathic. A high mean level of FVIII (above 190 IU/dL) was observed, supported by the reported elevated levels of FVIII in CTEPH patients [30, 31]. As an acute-phase protein, FVIII levels are elevated, for example, in infection, inflammation, and acute thrombosis, but high FVIII levels have an individual dose-dependent thrombotic role [32]. Also, in some patients, levels of CRP, another acute-phase reactant, were clearly elevated, in line with a proinflammatory and prothrombotic state [33]. We detected shortening of aPTT from index thrombosis to FXaI failure in three patients, also indicating an elevated risk of thrombosis [34].

Levels of D-dimer were significantly decreased from the index thrombosis and the start of FXaI treatment to FXaI failure. Three patients had high fibrinogen levels at the time of FXaI failure, but the mean value 3.6 g/L was within the reference range. Low D-dimer and high fibrinogen levels suggest impaired fibrinolysis when FXaI treatment fails. Future research is needed to study the connection between FXaI failure and impaired fibrinolysis. Anemia is associated with a bleeding tendency, but on the other hand iron-deficiency anemia is known to predispose individuals to thrombosis *via* hypoxia and distal endothelial activation and release of von Willebrand factor and FVIII [35, 36].

Although all our patients had major risk factors of thrombosis, VTE risk scores were high in only four patients when Padua and Caprini scores were used, and in five patients when HUH scores were used. In the other half of the patients VTE risk scores were low, possibly because none of the scores consider venous obstruction, such as in TOS. The high frequency of TOS in our cohort demands raising awareness of this underlying cause of VTE, its recurrence, and its influence on VTE risk scores. The difference between the score results can be explained by the fact that the HUH score includes high FVIII activity (>190 IU/dL), whereas the others do not. Moreover, the CTEPH score was high in only two patients, although CTEPH developed in six. Surprisingly, we observed a discrepancy between the VTE and CTEPH risk score results in many of the FXaI failure PE patients, suggesting that these scores do not necessarily align. In our series, the mean risk was high when using Caprini and HUH scores, but low with Padua scoring, which covers fewer risk factors. Notably, Caprini scoring is the only one that considers a family history of VTE. These observations may explain the differences between the mean results obtained in risk scoring.

This was a small retrospective case series study aiming at describing factors which predisposed VTE patients to FXaI failure, and its clinical consequences. The limitations of our study included a small sample size, and the lack of control group, whereby general conclusions cannot be drawn. However, these preliminary results generate future research topics for FXaI failure.

Taken together, for some patients, FXa inhibitors are not efficient enough to treat all forms of VTE. These patients may carry major risk factors, including mechanical venous obstruction and phospholipid antibodies, subjecting them to thrombosis reoccurrence. We should be able to recognize these high-risk patients and treat them with appropriate anticoagulants and mechanical intervention in the first place. Inefficient anticoagulation allows the thrombosis to organize into one that is highly resistant to endogenous fibrinolytic activity, leading to a need to subject the patients to mechanical or surgical treatment. A lesson to be learned from our study is that venous obstructions should be recognized and excluded as a risk factor of acute VTE, especially PE, its recurrence, and development of CTEPH. Also, some fibrinolysis deficiency may underline the VTE reoccurrence and its consequences, as D-dimer levels were unexpectedly low in FXaI failure.

## Data Availability

The data that support the findings of this study are available on request from the corresponding author (MK). The data are not publicly available since they contain information that could compromise the privacy of the research participants.
